# Efficacy and Safety of Tapinarof for Atopic Dermatitis: A Systematic Review and Meta-Analysis of Randomized Controlled Trials

**DOI:** 10.7759/cureus.91549

**Published:** 2025-09-03

**Authors:** Baraa A Saifi, Zyad A Saifi, Nancy Emmanuel

**Affiliations:** 1 Department of Medicine, Lebanese American University (LAU) Gilbert and Rose-Marie Chagoury School of Medicine, Byblos, LBN; 2 Faculty of Medicine, American University of Beirut Medical Center, Beirut, LBN; 3 Department of Dermatology, Faculty of Medicine, University of São Paulo, São Paulo, BRA

**Keywords:** aryl hydrocarbon receptor, atopic dermatitis, systematic review, tapinarof, topical therapy

## Abstract

This systematic review and meta-analysis evaluated the efficacy and safety of 1% tapinarof cream once daily (QD) in patients with mild-to-severe atopic dermatitis (AD). Adhering to Preferred Reporting Items for Systematic Reviews and Meta-Analyses (PRISMA) guidelines, we systematically searched PubMed, Embase, Cochrane, and ClinicalTrials.gov databases for placebo-controlled, randomized trials up to March 2025. Four studies reporting five randomized trials involving a total of 1191 patients met the inclusion criteria. Primary endpoints included Investigator Global Assessment (IGA) success and achievement of Eczema Area and Severity Index 75% (EASI-75) response. Secondary outcomes included improvement in Peak Pruritus Numeric Rating Scale (PP-NRS) scores and incidence of adverse events (AEs). After eight weeks, patients receiving tapinarof 1% QD were associated with significantly higher IGA success rates (risk ratio (RR) = 3.03, 95% CI: 1.94-4.74, p = 0.002), EASI-75 response rates (RR = 2.99, 95% CI: 1.94-4.60, p = 0.002), and clinically meaningful improvement in PP-NRS scores of ≥4 points (RR = 1.58, 95% CI: 1.10-2.26, p = 0.03) compared to placebo. Tapinarof also showed a modest but statistically significant increase in the overall incidence of adverse events (AEs) (RR = 1.39, 95% CI: 1.02-1.90, p = 0.04), while the rate of serious AEs was not significantly different between groups (RR = 1.98, 95% CI: 0.36-24.69, p = 0.31). In conclusion, tapinarof cream 1% QD demonstrated significant efficacy in treating mild-to-severe AD, with improvements in clinical signs and pruritus. It was generally well tolerated, supporting its use as a non-steroidal topical therapy in this patient population. Protocol registered in International Prospective Register of Systematic Reviews (PROSPERO) (CRD420251000807), with risk of bias assessed using the Cochrane risk-of-bias (RoB 2) tool and certainty of evidence appraised using the Grading of Recommendations Assessment, Development and Evaluation (GRADE) framework.

## Introduction and background

Atopic dermatitis (AD) is a chronic, relapsing inflammatory skin disease characterized by recurrent episodes of eczematous lesions, intense pruritus, xerosis, erythematous papules, excoriations, and lichenification [1-3]. AD involves a disruption in the skin barrier, which contributes to repeated cycles of inflammation and increases the risk of skin infections [4-6]. AD profoundly impairs patient quality of life, sleep, psychological well-being, and social functioning, often necessitating continuous management due to its recurrent nature [1].

Although AD is more common during childhood, the disease may persist into adulthood or even present later in life [1]. AD affects an estimated 15% to 20% of children and 1% to 3% of adults globally [1]. The steady rise in global prevalence makes AD an increasing public health concern [1]. 

There is no definitive cure for AD [7]. Current available treatments aim to reduce disease severity through pruritus control, infection prevention, and restoration of the skin barrier [7]. Because the disease tends to follow a remitting and relapsing course, long-term maintenance is essential. Newer systemic therapies and biologics have been designed to support prolonged disease control with favorable safety profiles, particularly in children [7].

Topical therapies are the mainstay of treatment used for induction and maintenance of remission in patients with AD [7]. Corticosteroids are first-line due to their potent anti-inflammatory effects [8,9]; however, prolonged use may lead to dermal atrophy, striae, telangiectasia, hirsutism, dyspigmentation, and cause withdrawal phenomenon [8,9]. More recently, nonsteroidal topical agents have become available, such as topical phosphodiesterase-4 (PDE4) inhibitors (e.g., crisaborole), topical calcineurin inhibitors (TCIs) (e.g., tacrolimus and pimecrolimus), and Janus kinase (JAK) inhibitors (e.g., ruxolitinib) [10]. TCI and topical PDE4 can cause local tolerability issues, such as application site pain, stinging, or burning [11-14]. For the treatment of severe AD, only one topical agent (TCI) is approved, limited to second-line, short-term application in immunocompetent individuals [12]. The only topical JAK inhibitor approved for AD carries a boxed warning and is restricted to individuals aged 12 and above, with treatment limited to no more than 20% of the body surface area (BSA) [14]. All approved topical calcineurin, JAK, and PDE4 inhibitors for AD are prescribed for twice-daily (BID) use, which can reduce patient adherence [11-14]. Given these limitations, there is still a need for effective and safe topical agents for the treatment of patients with mild to severe AD [10,15].

Tapinarof (VTAMA®, Dermavant Sciences, Inc., Morrisville, NC, GSK2894512, 5-[(E)-2-phenylethenyl] 2-(propan-2-yl) benzene-1,3-diol) is a topical nonsteroidal, aryl hydrocarbon receptor (AhR) agonist [16,17]. Tapinarof mainly works via ligand-dependent AhR activation, resulting in the downregulation of type 2 inflammatory cytokines (e.g., IL-4, IL-5, IL-13, IL-31), reducing skin inflammation [18]. Tapinarof increases the expression of important skin barrier proteins, including filaggrin, loricrin, involucrin, hornerin, and ceramides, promoting skin barrier recovery [18,19]. In addition, tapinarof was identified as a nuclear factor erythroid 2-related factor 2 pathway activator, which reduces oxidative stress and upregulates antioxidative enzymes expression [19]. In simple terms, tapinarof activates anti-inflammatory pathways and helps restore the skin barrier [19]. The FDA has already approved tapinarof cream (by Dermavant Sciences, Inc., in 2022) for the treatment of plaque psoriasis [16,17], a disease that shares key immunopathological features with AD, including Th2-mediated inflammation and compromised skin barrier integrity [4-6]. Phase 3 PSOARING clinical trials demonstrated that the drug was effective and well tolerated with sustained efficacy in patients with moderate to severe plaque psoriasis [16,17]. These findings suggest a potential role for tapinarof in the treatment of AD.

Tapinarof’s role in AD has been recently assessed in several clinical trials. Phases 2 and 3 randomized controlled trials (RCTs) confirmed improvement in disease severity scores with few reported adverse events (AEs) [20-23]. However, no comprehensive meta-analysis has yet synthesized these trials. This meta-analysis aims to assess the available clinical trials on the efficacy and safety of tapinarof in the treatment of patients with mild-to-severe AD aged two years and older, providing evidence-based information to facilitate clinical decision-making. Efficacy was evaluated based on improvements in Investigator Global Assessment (IGA) score, defined as achievement of a “clear” or “almost clear” IGA score with a ≥2-point improvement from baseline, Eczema Area and Severity Index 75% (EASI-75( response, defined as a ≥75% reduction in the EASI from baseline and Peak Pruritus Numeric Rating Scale (PP-NRS) improvement, corresponding to a ≥4-point reduction in itch severity. Safety was assessed through the incidence of AEs and serious AEs (SAEs).

## Review

Methods

Protocol and Registration

The prospective meta-analysis protocol has been registered in the International Prospective Register of Systematic Reviews (PROSPERO) under protocol CRD420251000807. The study was designed in compliance with the Preferred Reporting Items for Systematic Reviews and Meta-Analyses (PRISMA) reporting guideline [24].

Eligibility Criteria

We included studies based on the following eligibility criteria: (1) RCTs; (2) comparing topical tapinarof with placebo; (3) patients with mild-to-severe AD, defined as an EASI score of ≥ 5, a validated IGA for Atopic Dermatitis (vIGA-AD) score of ≥ 2, and an AD involvement of ≥ 5% of BSA; pediatric (aged two to 11), adolescents (aged 12-17) and adults (aged ≥ 18) patients were included; and (4) reporting at least one of the clinical outcomes of interest. We excluded studies that (1) compared tapinarof to other active treatments; (2) had an overlapping patient populations; (3) did not report any of the predefined primary outcomes of interest (IGA success and EASI-75); (4) were written in a language other than English; however, no eligible non-English studies were found; and (5) conference abstracts and preprints were excluded from analysis unless full peer-reviewed data were available. 

Search Strategy and Study Selection 

PubMed, Embase, Cochrane Central Register of Controlled Trials, and ClinicalTrials.gov databases were systematically searched from inception to March 2025 for studies published in English using the following terms: “tapinarof” or “gsk2894512” or “aryl hydrocarbon receptor agonist” and “atopic dermatitis.” References of included trials were evaluated for additional studies. Two reviewers (B.S. and Z.S.) independently screened studies using the inclusion and exclusion criteria and performed the data extraction. Discrepancies were resolved by discussion and consensus. Full search strings for each database are provided in the Appendices.

Data Collection Process and Data Items 

The following information was extracted: authors, publication year, study design, patient characteristics, country, interventions, number of cases, outcome measures, and treatment duration. Primary outcomes included are as follows: proportion of patients achieving IGA score for AD of IGA=0 (clear) or IGA=1 (almost clear) with a reduction from baseline of ≥2 points at week 8; and proportion of patients achieving at least a 75% reduction in EASI from baseline at week 8 (EASI-75 response). Secondary outcomes included are as follows: proportion of patients achieving a reduction of at least four points from baseline in PP-NRS at week 8 (NRS ≥ 4); AEs; and SAEs. Efficacy was evaluated based on improvements in IGA score, EASI-75 response, and PP-NRS, while safety was assessed through the incidence of AEs and SAEs.

Risk of Bias in Studies

Quality assessment of the included randomized studies was performed independently by two authors (B.S. and Z.S.) using the Cochrane risk-of-bias tool (RoB 2) [25].

Effect Measures

For dichotomous outcomes (EASI-75%, IGA response, PP-NRS, and AE rates), the risk ratio (RR) with 95% CI was calculated.

Synthesis Methods

The statistical analysis was conducted using RevMan 5.4 (The Cochrane Collaboration, Oxford, UK). In accordance with recommendations from the Cochrane Handbook for Systematic Reviews of Interventions [26], a random-effects (RE) model (Restricted Maximum Likelihood; Hartung-Knapp small-sample adjustment applied) was employed for all outcomes to account for anticipated clinical and methodological heterogeneity across the included studies. For rare events (SAEs), we used Mantel-Haenszel RRs with a treatment-arm continuity correction; results were confirmed in sensitivity analyses using a Generalized Linear Mixed Model (GLMM). For multi-arm trials, we pre-specified inclusion of the 1% once-daily (QD) arm to avoid double-counting.

When multiple outcome time-points were reported, we prioritized the eight-week endpoints common to all included trials. We extracted data using the intention-to-treat (ITT) population when available. Missing numerators or denominators were clarified by reviewing supplementary material.

Heterogeneity

The I² statistic, Cochrane’s Q test, and Tau-square were used to assess heterogeneity with I² thresholds of 25%, 50%, and 75% representing low, moderate, and high heterogeneity, respectively. 

Sensitivity and Subgroup Analyses

We performed leave-one-out sensitivity analyses for all outcomes to assess the stability of our findings. In this approach, each study was removed one at a time, and the meta-analysis was re-run using the remaining studies. A study was considered influential if its removal changed the pooled effect estimate or altered the statistical significance of the result.

Subgroup analyses by age, disease severity, anatomical site, and duration of therapy could not be performed due to insufficient stratified data.

Reporting Bias Assessment 

Funnel plots were not generated to assess potential publication bias, as the Cochrane Handbook recommends a minimum of 10 studies to reliably detect asymmetry [24].

Certainty of Evidence

The certainty of evidence for each outcome was evaluated using the Grading of Recommendations Assessment, Development and Evaluation (GRADE) approach. This involves assessing risk of bias, inconsistency, indirectness, imprecision, and publication bias, assigning a certainty rating (high, moderate, low, or very low) for each outcome. GRADE assessments were conducted using the GRADEpro Guideline Development Tool (GDT) software (Evidence Prime, Hamilton, Canada) [27].

Results

Study Selection and Baseline Characteristics

**Figure 1:**
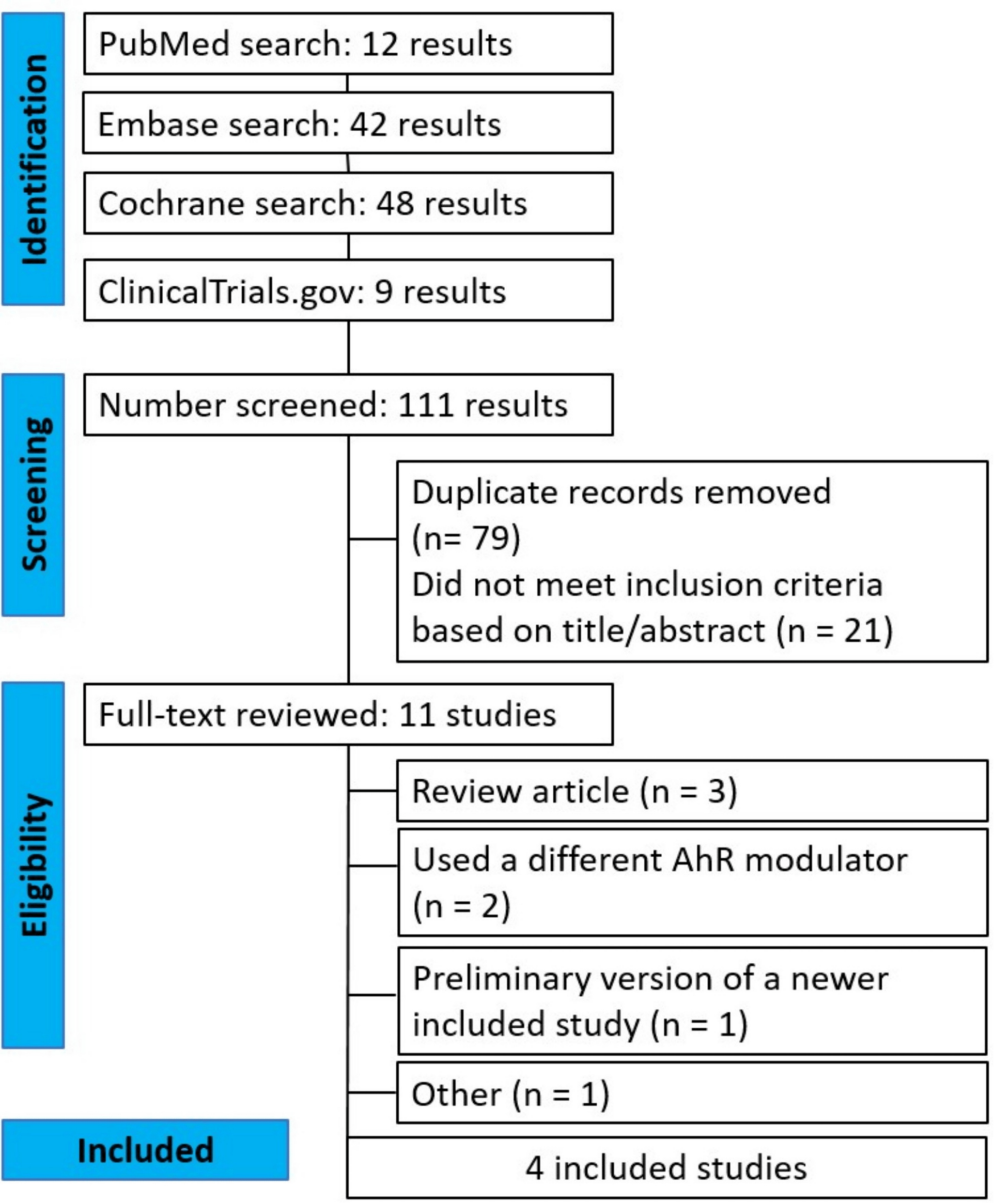
Preferred Reporting Items for Systematic Reviews and Meta-Analyses (PRISMA) flow diagram of study screening and selection. Four studies comprising five randomized controlled trials were included in the final analysis.

As detailed in Figure 1, our systematic search identified 111 potential articles. After removal of duplicate records and exclusion of articles that did not meet the inclusion criteria based on title and abstract review, 11 studies remained and underwent full-text revision. Ultimately, four studies were included encompassing five RCTs [20-23] with a total of 1191 patients diagnosed with mild to severe AD; 715 (60.0%) pediatric (aged 2-11), 260 (21.8%) adolescents (aged 12-17) and 216 (18.1%) adults (aged 18 and above), with an age range of 2 to 65 years old were included. From these, 767 (64.4%) patients were assigned to tapinarof 1% QD, and 424 (35.6%) patients were assigned to vehicle cream QD with a treatment period of 8 weeks. Based on baseline IGA scores, 89.8% of participants had moderate-to-severe disease, with only one small pediatric trial [22] enrolling patients with mild AD. Detailed study characteristics are presented in Table 1.

**Table 1:** Characteristics of studies included in meta-analysis. BSA = body surface area, EASI = eczema area and severity index, IGA = investigator’s global assessment, NA = not applicable, NM = not mentioned, NRS = numeric rating scale, QD = quaque die (once daily)

Reference (N)	Silverberg et al., 2024 (ADORING 1) (N = 407) [20]		Silverberg et al., 2024 (ADORING 2) (N = 406) [20]		Igarashi et al., 2025 (N = 121) [22]			Igarashi et al., 2024 (N = 214) [21]			Paller et al., 2021 (N = 83) [23]				
Intervention measures (n)	Tapinarof 1% QD	Vehicle QD	Tapinarof 1% QD	Vehicle QD	Tapinarof 1% QD	Tapinarof 0.5% QD	Vehicle QD	Tapinarof 1% QD	Vehicle QD	Tapinarof 1% BID	Tapinarof 1% QD	Tapinarof 0.5% BID	Tapinarof 0.5% QD	Vehicle BID	Vehicle QD
	(n = 270)	(n = 137)	(n = 271)	(n = 135)	(n = 41)	(n=40)	(n = 40)	(n = 144)	(n = 70)	(n = 40)	(n = 41)	(n = 43)	(n = 41)	(n = 42 )	(n = 40)
Study design	Phase 3, randomized controlled trial		Phase 3, randomized controlled trial		Phase 2, randomized controlled trial			Phase 3, randomized controlled trial			Phase 2b, randomized controlled trial				
Country	USA, Canada		USA, Canada		Japan			Japan			USA, Canada, Japan				
Age range, years	≥ 2		≥ 2		2-11			≥12			12-65				
Follow-up duration (weeks)	8		8		8			8			12				
Outcomes	IGA success, EASI-75, pruritus NRS, safety outcomes		IGA success, EASI-75, pruritus NRS, safety outcomes		IGA success, EASI-75, EASI 50, EASI90, % BSA affected, pruritus NRS, safety outcomes			IGA success, EASI-75, pruritus NRS, % BSA affected, Skindex-16 score, safety outcomes			IGA success, EASI-75, pruritus NRS score, % BSA affected, EASI90, POEM (patient-reported outcomes), safety outcomes				
Age, mean (SD), years	15.6 (16.62)	15.6 (16.49)	16.4 (16.24)	16.7 (16.05)	7.1 (3.1)	7.0 (2.6)	6.4 (2.8)	31.7 (12.3)	35.4 (13.0)	28.5 (13.9)	31.6 (15.7)	29.0 (15.9)	29.3 (14.0)	27.9 (14.7)	29.4 (15.2)
Male, n (%)/female, n (%)	130(48.1)/140(51.9)	66 (48.2)/71 (51.8)	117 (43.2)/154 (56.8)	58 (43.0)/77 (57.0)	23 (57.5)/18 (42.5)	21 (52.5)/19 (47.5)	22 (53.7)/18 (46.3)	105 (72.9)/39 (27.1)	46 (65.7)/24 (34.3)	22 (55.0)/18 (45.0)	17 (41.0)/24 (59.0)	26 (60.0)/16 (40.0)	19 (46.0)/22 (54.0)	19 (45.0)/23 (55.0)	23 (58.0)/17 (42.0)
Weight, mean (SD), kg	46.7 (27.3)	47.7 (27.7)	51.5 (29.1)	54.0 (32.0)	26.3 (10.7)	24.7 (8.3)	23.1 (9.2)	64.1 (13.4)	64.0 (12.1)	NM	NM	NM	NM	NM	NM
BMI, mean (SD), kg/m^2^	21.4 (6.3)	22.1 (6.6)	22.7 (7.5)	23.3 (8.3)	17.2 (2.5)	16.7 (2.4)	16.5 (2.1)	23.0 (3.8)	23.6 (3.5)	NM	NM	NM	NM	NM	NM
IGA-AD score, mean (SD)	3.0 (0.3)	3.1 (0.3)	3.2 (0.4)	3.2 (0.4)	2.6 (0.6)	2.6 (0.5)	2.5 (0.5)	3.1 (0.3)	3.1 (0.3)	3.1 (0.2)	3.1 (0.3)	3.1 (0.3)	3.1 (0.3)	3.1 (0.4)	3.1 (0.3)
IGA score, n (%)															
2 (Mild)	NA	NA	NA	NA	19 (47.5)	19 (47.5)	20 (48.8)	NA	NA	NM	NM	NM	NM	NM	NM
3 (Moderate)	244 (90.4)	122 (89.1)	228 (84.1)	113 (83.7)	20 (50.0)	20 (50.0)	19 (46.3)	132 (91.7)	64 (91.4)	NM	NM	NM	NM	NM	NM
4 (Severe)	26 (9.6)	15 (10.9)	43 (15.9)	22 (16.3)	1 (2.5)	1 (2.5)	2 (4.9)	12 (8.3)	6 (8.6)	NM	NM	NM	NM	NM	NM
EASI score, mean (SD)	12.2 (5.0)	12.9 (5.6)	13.5 (5.6)	13.1 (4.7)	11.7 (4.7)	11.2 (4.1)	11.8 (4.6)	15.9 (4.3)	15.3 (3.2)	9.8 (5.2)	10.9 (6.1)	13.1 (6.7)	11.4 (5.8)	11.1 (5.9)	11.1 (5.8)
BSA affected, mean (SD), %	16.5 (8.7)	17.7 (9.5)	17.1 (8.7)	15.8 (7.9)	20.4 (6.7)	20.2 (6.7)	11.8 (4.6)	21.3 (6.4)	21.4 (6.1)	14.8 (8.7)	18.7 (11.0)	19.7 (10.5)	17.6 (9.9)	14.5 (9.1)	16.0 (10.3)

Sensitivity Analyses

Leave-one-out analyses confirmed the robustness of most outcomes. For IGA success, significance was lost only when both ADORING trials were excluded, which together contributed 82.7% of the total weight, explaining their strong influence on the pooled result. Similarly, for PP-NRS ≥4, removing both ADORING studies (59.3% weight) also led to a loss of significance. In contrast, EASI-75 remained statistically significant regardless of which study was removed, indicating a stable and consistent effect. For AEs, significance was lost with the removal of any study except Igarashi et al. [22], likely due to small sample sizes and low event rates.

Risk of Bias Within Studies

**Figure 2:**
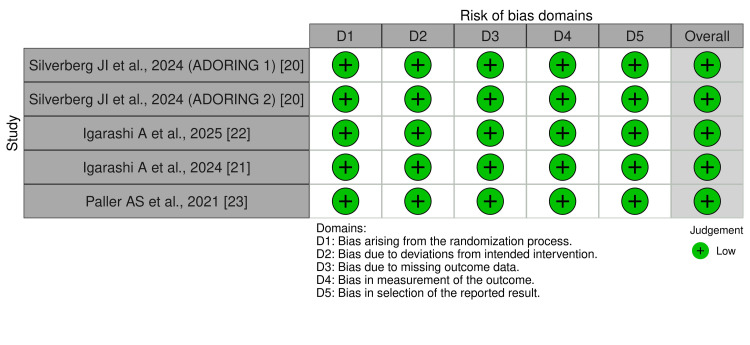
Risk of bias (RoB 2) summary for randomized studies. All five trials demonstrated low risk of bias across all five domains: bias arising from the randomization process (D1), bias due to deviations from intended interventions (D2), bias due to missing outcome data (D3), bias in measurement of the outcome (D4), and bias in selection of the reported result (D5). Overall, all studies were judged to have a low risk of bias.

For quality assessment, the Cochrane RoB 2 tool was used [25,28]. All five trials [20-23] were randomized using random sequence generation, double-blinded, and provided detailed data for assessment. Each trial clearly reported methods for allocation concealment and randomization, addressed withdrawals and attrition, and demonstrated no concerns across the five bias domains: randomization process, deviations from intended interventions, missing outcome data, measurement of the outcome, and selection of the reported result. Overall, all studies were judged to be at low risk of bias (Figure 2).

Efficacy

In terms of efficacy, we analyzed efficiency measures, including EASI scores, IGA, EASI-75, and pruritus NRS scores, after eight weeks of treatment. In line with Cochrane guidelines, a RE model was applied to all outcome analyses [26].

**Figure 3:**
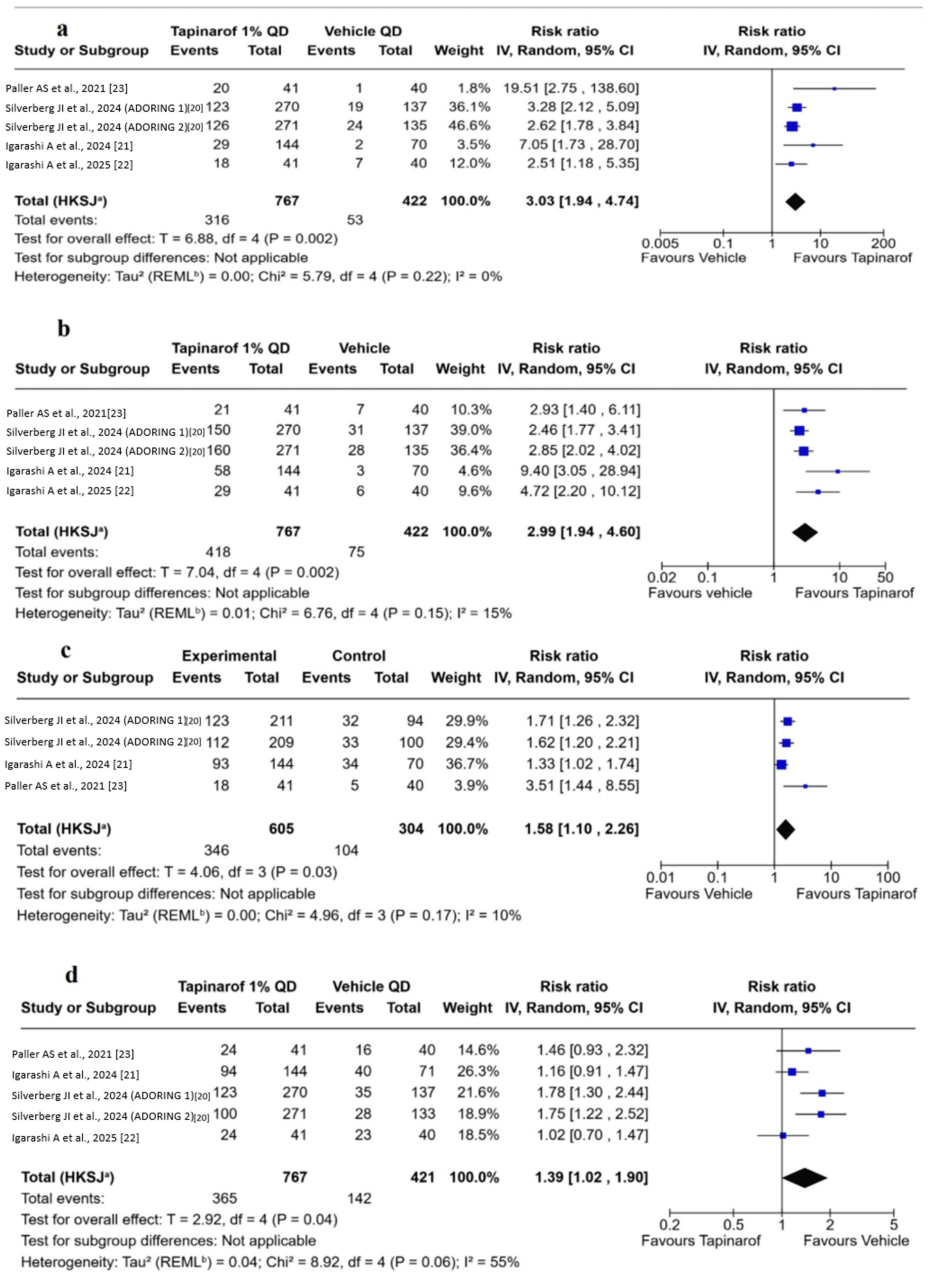
Forest plots for primary and secondary outcomes after eight weeks of treatment. a: Patients applying Tapinarof daily were three times more likely to achieve Investigator Global Assessment (IGA) success compared to vehicle. b: Tapinarof significantly increased the proportion of patients achieving Eczema Area and Severity Index 75% (EASI-75) compared to vehicle. c: Tapinarof significantly improved pruritus severity, with more patients achieving ≥4-point PP-NRS reduction compared to vehicle. d: The overall incidence of AEs was modestly but significantly higher in the Tapinarof group than in the vehicle group. Squares represent 95% confidence intervals (CIs), and diamonds represent pooled proportions.

IGA success/investigator’s global assessment 0/1: All included trials [20-23] provided data on the proportion of patients achieving IGA success, defined as an IGA score of 0 (clear) or 1 (almost clear) with at least a two-point improvement from baseline. The pooled analysis revealed that patients treated with tapinarof 1% cream QD achieved significantly higher IGA success rates at eight weeks compared to the placebo group, with a pooled RR of 3.03 (95% CI: 1.94-4.74; p = 0.002). Heterogeneity across studies was low (χ² = 5.79; df = 4; p = 0.22; I² = 0%; τ²= 0.00) (Figure 3). This corresponds to an absolute risk difference of 28.6% (41.2% with tapinarof vs. 12.6% with vehicle), yielding a number needed to treat (NNT) of 3.5 to achieve one additional IGA success with tapinarof compared to placebo.

EASI-75 response: All included trials [20-23] provided data on the proportion of patients achieving EASI-75 response, defined as patients achieving at least a 75% reduction in EASI from baseline. The pooled analysis revealed that patients treated with tapinarof 1% QD were significantly more likely to achieve EASI-75 compared to placebo after eight weeks of treatment, with a pooled RR of 2.99 (95% CI: 1.94 to 4.60; p = 0.002). The heterogeneity among the studies was low (χ² = 6.76; df = 4; p = 0.15; I² = 15%; τ² = 0.01) (Figure 3). Based on pooled event rates (54.5% with tapinarof vs. 17.8% with vehicle), this corresponds to an absolute risk reduction of 36.7% and an NNT of approximately 2.7.

PP-NRS improvement/NRS ≥ 4: Four trials [20-23] reported data on PP-NRS improvement, defined as the proportion of patients achieving a clinically meaningful reduction (≥4 points) from baseline, with a total of 909 patients included in the analysis. The pooled analysis revealed a significantly higher proportion of patients experiencing clinically meaningful improvement in pruritus at 8 weeks with tapinarof 1% QD compared to placebo, with a pooled RR of 1.58 (95% CI: 1.10 to 2.26; p = 0.03). Heterogeneity among the included trials was low (χ² = 6.76, df = 4; p = 0.15; I² = 15%; τ² = 0.00) (Figure 3).

Safety

AEs: All included trials reported data on AE rates [20-23]. The pooled analysis indicated a moderately increased risk of the frequency of the total AEs at eight weeks in the tapinarof group compared to placebo, with a pooled RR of 1.39 (95% CI: 1.02 to 1.90; p = 0.04). Moderate heterogeneity was noted among studies (χ² = 8.92, df = 4; p = 0.06; I² = 55%; τ² = 0.04; prediction interval 0.75 to 2.56) (Figure 3). A summary of all reported AEs can be found in Table 2.

**Table 2:** Detailed adverse events of the included studies. N/A = not applicable

Reference (N)	Silverberg et al., 2024 (ADORING 1) (N = 407) [20]		Silverberg et al., 2024 (ADORING 2) (N = 406) [20]		Igarashi et al., 2025 (N = 121) [22]		Igarashi et al., 2024 (N = 214) [21]		Paller et al., 2021 (N = 83) [23]	
Intervention measures (n)	Tapinarof 1% QD	Vehicle QD	Tapinarof 1% QD	Vehicle QD	Tapinarof 1% QD	Vehicle QD	Tapinarof 1% QD	Vehicle QD	Tapinarof 1% QD	Vehicle QD
	(n = 270)	(n = 137)	(n = 271)	(n = 135)	(n = 41)	(n = 40)	(n = 144)	(n = 70)	(n = 41)	(n = 40)
AE category (%)										
Any AEs	123 (45.6%)	35 (25.5%)	100 (36.9%)	28 (21.1%)	24 (58.5%)	23 (57.5%)	94 (65.3%)	30 (42.3%)	24 (59%)	16 (40%)
Serious AEs	3 (1.1%)	0 (0%)	2 (0.7%)	0 (0%)	0 (0%)	0 (0%)	0 (0%)	0 (0%)	0 (0%)	0 (0%)
Severe AEs	N/A	N/A	N/A	N/A	0 (0%)	0 (0%)	0 (0%)	0 (0%)	0 (0%)	0 (0%)
Treatment-related AEs	34 (12.6%)	9 (6.6%)	32 (11.8%)	9 (6.8%)	7 (17.1%)	10 (25.0%)	55 (38.2%)	12 (16.9%)	22 (54%)	15 (38%)
AEs leading to discontinuation	5 (1.9%)	5 (3.6%)	4 (1.5%)	4 (3.0%)	1 (2.4%)	7 (17.5%)	9 (6.3%)	9 (12.7%)	0 (0%)	2 (5%)
Application site irritation	N/A	N/A	N/A	N/A	3 (7.3%)	5 (12.5%)	2 (1.4%)	2 (2.8%)	N/A	N/A
Nasopharyngitis	13 (4.8%)	7 (5.1%)	4 (1.5%)	1 (0.8%)	4 (9.8%)	1 (2.5%)	8 (5.6%)	2 (2.8%)	5 (12%)	3 (8%)
Headache	19 (7.0%)	3 (2.2%)	4 (1.5%)	0 (0%)	3 (7.3%)	0 (0%)	23 (16.0%)	1 (1.4%)	1 (2%)	2 (5%)
Folliculitis	22 (8.1%)	1 (0.7%)	22 (8.1%)	2 (1.5%)	N/A	N/A	17 (11.8%)	1 (1.4%)	8 (20%)	0 (0%)
Acne	N/A	N/A	N/A	N/A	N/A	N/A	14 (9.7%)	1 (1.4%)	0 (0%)	1 (2%)
Contact dermatitis	4 (1.5%)	3 (2.2%)	3 (1.1%)	2 (1.5%)	1 (2.4%)	3 (7.5%)	5 (3.5%)	2 (2.8%)	N/A	N/A
Pyrexia	N/A	N/A	N/A	N/A	3 (7.3%)	1 (2.5%)	1 (0.7%)	0 (0%)	N/A	N/A
Upper respiratory tract infection/inflammation	N/A	N/A	N/A	N/A	3 (7.3%)	1 (2.5%)	N/A	N/A	2 (5%)	4 (10%)
COVID-19	N/A	N/A	N/A	N/A	0 (0%)	2 (5.0%)	3 (2.1%)	1 (1.4%)	N/A	N/A
Atopic dermatitis exacerbation	N/A	N/A	N/A	N/A	0 (0%)	6 (15.0%)	7 (4.9%)	11 (15.5%)	0 (0%)	5 (13%)
Oral herpes	N/A	N/A	N/A	N/A	2 (4.9%)	0 (0%)	0 (0%)	0 (0%)	N/A	N/A
Skin papilloma	N/A	N/A	N/A	N/A	N/A	N/A	4 (2.8%)	0 (0%)	N/A	N/A
Rhinitis	N/A	N/A	N/A	N/A	0 (0%)	2 (5.0%)	N/A	N/A	N/A	N/A

**Figure 4:**
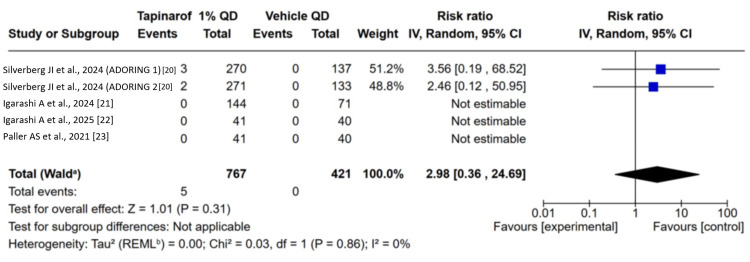
Forest plot for serious adverse events. No significant difference between the tapinarof group and the placebo group in the incidence of serious adverse events (SAEs).

SAEs: All included trials [20-23] reported data on the incidence of SAE rates. The pooled analysis indicated no difference between groups in the incidence of SAE rates between the tapinarof group compared to placebo, with a pooled RR of 1.98 (95% CI: 0.36 to 24.69; p = 0.31). Low heterogeneity was noted among studies (χ² = 80.03; df = 1; p = 0.86; I² = 0%; τ² = 0.00) (Figure 4).

Reporting Bias

As fewer than 10 studies were included in each meta-analysis, funnel plots and formal tests for publication bias were not performed, per the Cochrane Handbook recommendations [24]. 

Certainty of Evidence (GRADE Assessment)

Our findings provide high-certainty evidence regarding the efficacy outcomes (IGA success, EASI-75, pruritus improvement), while the certainty for AE outcomes was moderate due to moderate heterogeneity across studies (I² = 55%). No other outcomes required downgrading. Certainty ratings were determined using the GRADE framework, with downgrades applied when appropriate based on factors such as imprecision (e.g., wide confidence intervals or limited sample size) and indirectness (e.g., variability in outcome measurement or population characteristics. A summary of findings for the main efficacy and safety outcomes, along with the certainty of evidence assessed using the GRADE approach, is provided in Table 3.

**Table 3:** Summary of findings table for tapinarof vs. vehicle in mild to severe atopic dermatitis. *The risk in the intervention group (and its 95% confidence interval) is based on the assumed risk in the comparison group and the relative effect of the intervention (and its 95% CI). ^a^High heterogeneity (I² > 50%) downgrade by one level for inconsistency. EASI-75 = Eczema Area and Severity Index 75%, IGA = Investigator Global Assessment, PP-NRS = Peak Pruritus Numeric Rating Scale, QD = quaque die (once daily), RCT = randomized controlled trial, RR = risk ratio GRADE Working Group grades of evidence are as follows. High certainty: We are very confident that the true effect lies close to that of the estimate of the effect. Moderate certainty: We are moderately confident in the effect estimate; the true effect is likely to be close to the estimate of the effect, but there is a possibility that it is substantially different. Low certainty: Our confidence in the effect estimate is limited; the true effect may be substantially different from the estimate of the effect. Very low certainty: We have very little confidence in the effect estimate; the true effect is likely to be substantially different from the estimated effect.

Outcomes	Anticipated absolute effects*(95% CI)		Relative effect (95% CI)	No. of participants (studies)	Certainty of the evidence (GRADE)	Comments
	Risk with Vehicle QD	Risk with Tapinarof 1% QD				
IGA Success	126 per 1000	381 per 1000 (244 to 595)	RR 3.03 (1.94 to 4.74)	1189 (5 RCTs)	⨁⨁⨁⨁ High	Tapinarof 1% QD results in large increase in IGA Success rates [20-23]
EASI-75 Response	178 per 1000	531 per 1000 (345 to 818)	RR 2.99 (1.94 to 4.60)	1189 (5 RCTs)	⨁⨁⨁⨁ High	Tapinarof 1% QD results in large increase in EASI-75 Response rates [20-23]
PP-NRS improvement	342 per 1000	541 per 1000 (376 to 773)	RR 1.58 (1.10 to 2.26)	909 (4 RCTs)	⨁⨁⨁⨁ High	Tapinarof 1% QD results in a moderate increase in PP-NRS improvement rates [20,21,23]
Adverse events	337 per 1000	469 per 1000 (344 to 641)	RR 1.39 (1.02 to 1.90)	1188 (5 RCTs)	⨁⨁⨁◯ Moderate^a^	Tapinarof 1% QD probably results in a moderate increase in adverse events [20-23]

Discussion

Summary of Main Findings

According to the data we collected, we selected IGA treatment success rates, EASI-75 response, and PP-NRS improvement rates to assess the treatment efficacy of tapinarof cream. The IGA and EASI-75 scores evaluate primarily localized lesions, assessing disease severity based on eczema characteristics such as erythema, excoriation, induration, and lichenification. In contrast, the PP-NRS score evaluates itch intensity, a key symptom impacting the quality of life in AD patients.

In this systematic review and meta-analysis of five RCTs and 1191 patients, we compared topical tapinarof cream 1% QD with placebo in patients with mild-to-severe AD after eight weeks of treatment. The main findings were as follows: (1) patients applying tapinarof daily were three times more likely to achieve IGA success and EASI-75 response compared to placebo (RR 3.03, 95% CI 1.94-4.74, p = 0.002; RR 2.99, 95% CI 1.94-4.60, p = 0.002, respectively); (2) treatment with tapinarof resulted in significant improvement in pruritus measured by PP-NRS score (RR 1.58, 95% CI 1.10-2.26, p = 0.03); and (3) tapinarof exhibited a favorable safety profile, although associated with a slight but statistically significant increase in mild-to-moderate AEs compared with placebo (RR 1.39, 95% CI 1.02-1.90, p = 0.04).

Comparison With Existing Literature

An initial formulation of tapinarof, known as WBI-1001, developed by Welichem Biotech (Burnaby, Canada), the former asset owner, has been investigated in prior clinical trials, which provided preliminary evidence of the drug’s efficacy and safety in AD [29]. This meta-analysis particularly focuses on the current formulation, VTAMA® cream (tapinarof; GSK2894512) developed by Dermavant Sciences, Inc. and approved for plaque psoriasis [30], as it is closest to clinical practice. To maintain consistency and clinical applicability, studies involving the earlier WBI-1001 formulation were not included in our meta-analysis.

A recent systematic review and meta-analysis by de Farias Santos et al. [31] reported similar efficacy and safety outcomes for tapinarof in AD. Their analysis incorporated benvitimod (WBI-1001), the earlier formulation of the molecule [29], showing statistically significant improvements in IGA success rates and EASI-75 scores, with sensitivity analysis excluding benvitimod not altering significance [31]. In contrast, our analysis focused exclusively on the currently approved VTAMA® formulation of tapinarof [30] and included one additional pediatric study [22], further reinforcing the efficacy and safety profile of the currently approved formulation across the pediatric population. Both analyses identified folliculitis and headache as common AEs [31]. Interestingly, de Farias Santos et al. found these events to be statistically significant (RR 6.21; p < 0.001 and RR 3.24; p = 0.025, respectively) [31], although headache causality in relation to tapinarof cream remains unclear [31]. Notably, their review did not evaluate PP-NRS (PP-NRS ≥ 4), a key symptom burden in AD. Our study addresses this gap by providing pooled estimates for antipruritic efficacy.

Pruritus relief was observed from as early as two weeks of treatment initiation [21,23]. The rapidity of symptom improvement, particularly for pruritus, is clinically relevant and directly affects patient adherence and satisfaction [21]. However, specific timing for initial relief was not consistently reported across all trials [20,22]. It is recommended that future randomized trials assess and report time-to-relief outcomes to better inform clinical decision-making.

Tapinarof treatment demonstrated an acceptable safety profile. Although patients receiving Tapinarof experienced a higher frequency of AEs compared with placebo, the events were typically mild, well-tolerated, and rarely led to treatment discontinuation. The most commonly reported AEs included folliculitis (9.0%), headache (6.5%), nasopharyngitis (4.4%), acne (1.8%), and application site irritation (0.7%) (Table 2). These AE are consistent with AE of other topical therapies for AD, such as topical corticosteroids and calcineurin inhibitors [7]. The acne and folliculitis lesions primarily presented as inflammatory papules and pustules, and all reported cases were non-serious. Headache events generally occurred early in treatment and were of short duration [21]. There were a few cases with asymptomatic elevations in aminotransferase levels; the events were transient and did not necessitate discontinuation of treatment [20]. Regarding serious AEs, there was no significant difference between the tapinarof and placebo groups.

While our meta-analysis primarily assessed outcomes at eight weeks, extended-duration studies further support tapinarof cream’s favorable long-term safety. The Japanese phase 3 trial by Igarashi et al. [21] assessed treatment with tapinarof up to 52 weeks, showing sustained efficacy and stable safety without emergent concerns. Likewise, the ADORING 3 trial assessed tapinarof 1% cream QD for up to 48 weeks in their adult and pediatric patients down to two years of age, showing consistent safety outcomes and the absence of new safety concerns with prolonged use [32].

This study design only included trials using tapinarof 1% QD regimen, as a pooled analysis of the other regimes (1% BID and 0.5% QD and BID) could not be performed due to insufficient data. In the initial phase 2 dose-ranging study [23,30], multiple doses and regimens were compared. The 1% QD regimen exhibited efficacy superior to that of the 0.5% QD regimen and exhibited comparable efficacy to 1% BID regimen (IGA success rate 46% vs. 53%, respectively) while maintaining a lower incidence of AEs (54% vs. 70%, respectively) [23,33]. Subsequently, trials that were published after Paller et al. adopted the 1% QD regimen across pediatric [20,22] and adult populations [20-23]. While a pediatric trial compared tapinarof 0.5% QD to 1% QD [22], the 1% QD regimen has remained the predominant choice across the adult population.

Strengths and Limitations

This meta-analysis provides valuable insights into the efficacy and safety of tapinarof in AD, focusing exclusively on the approved VTAMA® 1% QD formulation. It incorporates recent pediatric data and evaluates PP-NRS, underscoring the drug’s impact on clinically meaningful symptom relief. The study followed PRISMA 2020 guidelines with rigorous methodology and GRADE assessment, enhancing the reliability and clinical relevance of its findings.

Despite these strengths, several limitations should be acknowledged. The overall number of included studies was limited, with relatively small sample sizes in some trials, which may affect the generalizability of our findings. Subgroup analyses by age, disease severity, anatomical site, and duration of therapy could not be performed due to insufficient stratified data. Only English-language studies were included, which may have also introduced language bias. Although current findings are promising, tapinarof is a relatively new therapeutic option for AD; thus, further RCTs, particularly those comparing tapinarof to active comparators, are needed to comprehensively assess its long-term efficacy and safety. Finally, real-world data on adherence, cost-effectiveness, and long-term outcomes are limited and should be addressed in future studies.

Clinical Implications and Research Needs

Tapinarof offers distinct advantages that could enhance its clinical utility in real-world practice. Tapinarof cream represents a nonsteroidal, QD topical option, addressing adherence barriers typically associated with the BID regimens required for topical PDE4, calcineurin, and JAK inhibitors [10-14]. Additionally, tapinarof exhibits fewer long-term safety concerns compared to topical corticosteroids and has demonstrated a favorable tolerability profile. Therefore, tapinarof may effectively bridge a therapeutic gap, particularly benefiting patients with significant steroid phobia and those showing inadequate response to standard topical therapies and who are still not optimal candidates for systemic treatments.

To further clarify pediatric dosing and safety, an additional Japanese phase 3 trial is planned, assessing specifically the 0.5% tapinarof cream formulation in pediatric patients aged two to 11 years [22]. The lower concentration was chosen based on results from the Japanese pediatric phase 2 trial, where tapinarof 0.5% QD demonstrated numerically higher efficacy (EASI-75 response: 77.5% vs. 70.7%) and fewer AEs compared to the 1% concentration [22]. Therefore, optimal pediatric dosing remains uncertain, warranting further investigation.

## Conclusions

In conclusion, 1% tapinarof cream applied once daily for eight weeks reduced disease severity, improved clinical outcomes (IGA success, EASI-75 response, and pruritus), and demonstrated acceptable tolerability in patients with mild-to-severe AD. The composite of clinical improvement (IGA success or EASI-75) and patient-reported outcomes (pruritus NRS) is consistently improved by tapinarof cream across multiple patient subgroups, including pediatric and adult populations. Finally, the incidence of AEs with tapinarof cream appears mild and acceptable. These findings support the use of tapinarof cream 1% once daily as a potential new therapeutic option in the management of AD.

## Appendices

Search strategy for each database up to the 1st of March 2025

Pubmed

("Tapinarof" OR "GSK2894512" OR "Aryl Hydrocarbon Receptor Agonist") AND ("Atopic

Dermatitis"[ti]) AND (randomized controlled trial[pt] OR controlled clinical trial[pt] OR clinical trials as topic[mesh:noexp] OR trial[ti] OR random*[tiab] OR placebo*[tiab])

Embase

('tapinarof'/exp OR 'tapinarof':ti,ab OR 'gsk2894512':ti,ab OR 'aryl hydrocarbon receptor agonist':ti,ab) AND ('atopic dermatitis'/exp OR 'atopic dermatitis':ti,ab) AND 'randomized controlled trial'/de

Cochrane

("Tapinarof" OR "GSK2894512" OR “Aryl Hydrocarbon Receptor Agonist”)  AND  ("Atopic Dermatitis")

ClinicalTrials.gov

(tapinarof OR gsk2894512 OR aryl hydrocarbon receptor agonist) AND atopic dermatitis
